# *OBE3* and *WUS* Interaction in Shoot Meristem Stem Cell Regulation

**DOI:** 10.1371/journal.pone.0155657

**Published:** 2016-05-19

**Authors:** Ta-Fang Lin, Shunsuke Saiga, Mitsutomo Abe, Thomas Laux

**Affiliations:** 1 BIOSS Centre for Biological Signalling Studies, Faculty of Biology, University of Freiburg, Schaenzlestrasse 1, 79104, Freiburg, Germany; 2 Laboratory of Biochemistry, Wageningen University, Dreijenlaan 3, 6703 HA, Wageningen, The Netherlands; 3 Department of Biological Sciences, Graduate School of Science, The University of Tokyo, 7-3-1 Hongo, Bunkyo-ku, Tokyo, 113–0033, Japan; Universidad Miguel Hernández de Elche, SPAIN

## Abstract

The stem cells in the shoot apical meristem (SAM) are the origin of all above ground tissues in plants. In *Arabidopsis thaliana*, shoot meristem stem cells are maintained by the homeobox transcription factor gene *WUS* (*WUSCHEL*) that is expressed in cells of the organizing center underneath the stem cells. In order to identify factors that operate together with *WUS* in stem cell maintenance, we performed an EMS mutant screen for modifiers of the hypomorphic *wus-6* allele. We isolated the *oberon3-2* (*obe3-2*) mutant that enhances stem cell defects in *wus-6*, but does not affect the putative null allele *wus-1*. The *OBE3* gene encodes a PHD (Plant Homeo Domain) protein that is thought to function in chromatin regulation. Single mutants of *OBE3* or its closest homolog *OBE4* do not display any defects, whereas the *obe3-2 obe4-2* double mutant displays broad growth defects and developmental arrest of seedlings. Transcript levels of *WUS* and its target gene in the stem cells, *CLAVATA3*, are reduced in *obe3-2*. On the other hand, *OBE3* and *OBE4* transcripts are both indirectly upregulated by ectopic *WUS* expression. Our results suggest a positive feedback regulation between *WUS* and *OBE3* that contributes to shoot meristem homeostasis.

## Introduction

Postembryonic growth and iterative organ formation of higher plants rely on the activity of pluripotent stem cells in organogenic centers, the meristems. The shoot meristem that will give rise to the above ground organs has been extensively studied in the model plant *Arabidopsis thaliana*. The homeodomain transcription factor WUS is expressed in the organizing center (OC) underneath the stem cells [1] where it directly represses cytokinin response inhibitors [2] and, after moving into the overlying stem cells [3, 4], represses cell differentiation and activates expression of the signal peptide CLV3 [4–6]. CLV3 in turn represses *WUS* transcription via CLV1/CLV2-CRN receptor-like kinases to delimit the size of the OC [6–8]. This negative feedback loop balances stem cell maintenance and differentiation [7]. The WUS/CLV3 loop also functions to maintain stem cells of the floral meristems [6, 7]. In contrast to the indeterminate shoot meristem, WUS in the determinate floral meristem also activates the gene encoding the MADS domain protein AGAMOUS (AG) that in turn terminates WUS expression and thus floral meristem growth [9–11]. In addition to its function in stem cell regulation, WUS is also required for the development of the female and male gametes [12–14]. However, CLV3 signaling does not appear to be targeted by WUS in these cases.

Although in the recent years, many studies identified further components affecting WUS/CLV3 homeostasis [3, 15–23], how WUS maintains stem cells remains enigmatic.

In order to find hitherto undiscovered factors involved in the WUS-mediated stem cell regulation, we used a sensitized mutant screen for genetic modifiers of the hypomorphic *wus-6* allele [21, 24]. Here we report the isolation of the *wus enhancer 9* (*wen9*) mutant that enhances stem cell defects in *wus-6*. We show by positional cloning that *wen9* is an allele of the *OBE3* gene, and characterize its function together with its closest homologue *OBE4* in the shoot meristem.

## Results

### *wen9* enhances inflorescence shoot meristem defects of *wus-6*

**Fig 1:**
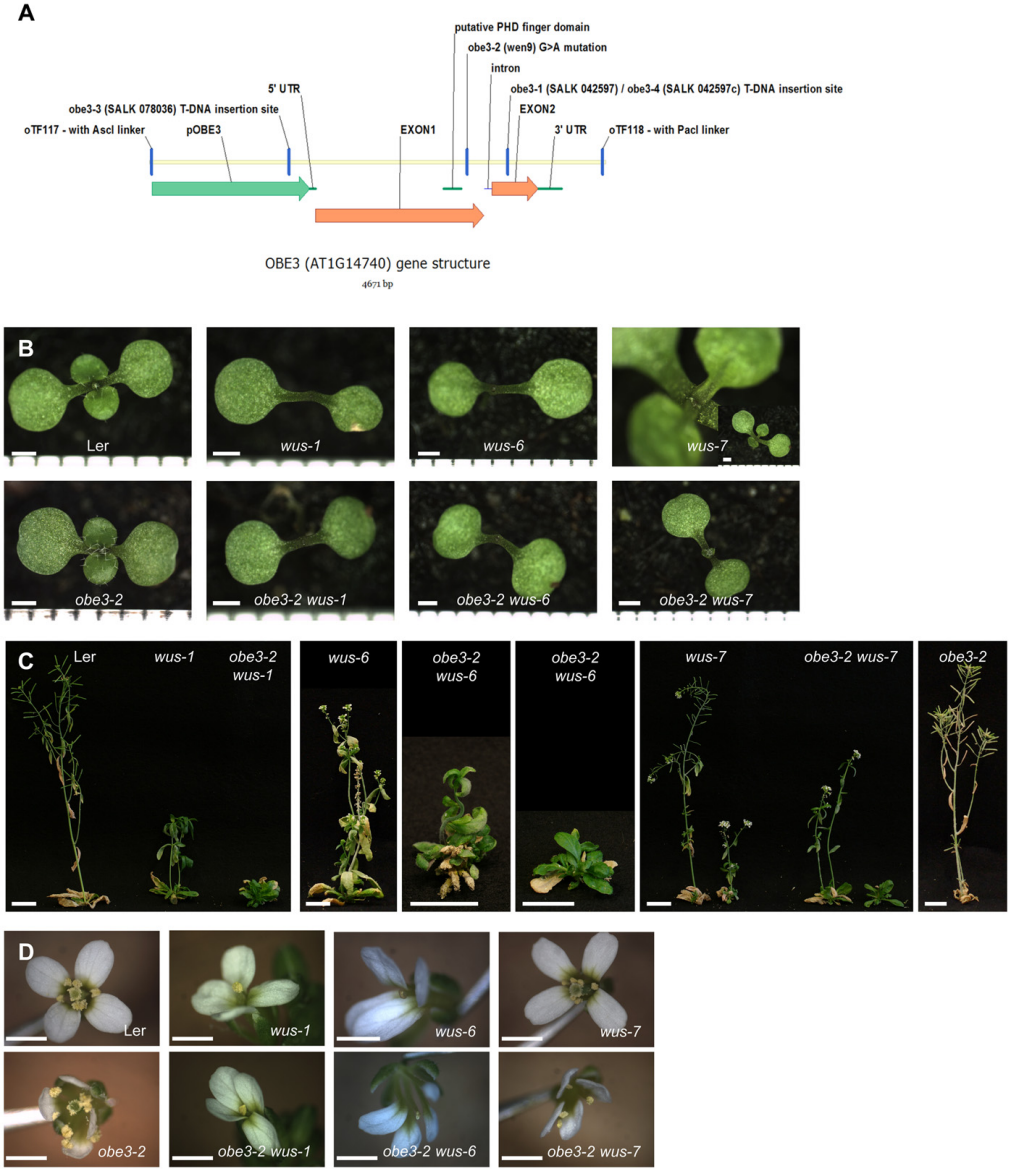
*OBE3* gene structure and mutant phenotypes. (A) Structure of the *OBE3* gene. The upstream region used for the complementation is shown in green. (B-D) Phenotypes of the denoted genotypes of 10-day-old seedlings (B), shoots (C), and flowers (D). Scale bars: 1 mm (B, D), 2 cm (C).

The putative null allele *wus-1* causes premature termination of stem cells in the primary shoot meristem during embryogenesis, resulting in a flat apex of partially differentiated cells at the seedling stage [6]. Consequently, seedlings lack any true leaves at 10 days after germination (Fig 1B). Postembryonically initiated shoot meristems terminate after the formation of a few leaves, resulting in a stop-and-go phenotype (Fig 1C), and the seldom formed floral meristems give rise to 4 sepals, 4 petals, and a single stamen before premature termination (Fig 1D). The intermediate *wus-6* allele causes reduced *WUS* expression levels, and the primary seedling shoot meristem and floral meristem prematurely terminate indistinguishably to *wus-1* (Fig 1B and 1D; [21, 24]. In contrast to *wus-1*, however, postembryonically initiated *wus-6* shoot meristems grow indeterminately and give rise to many floral meristems (Fig 1C; Tables 1 and 2). The *wus-7* allele carries a missense mutation in the homeodomain and represents the weakest known *wus* allele [25]. *wus-7* seedlings form several rosette leaves before the primary shoot meristem terminates (Fig 1B) and axillary shoot meristems form indeterminate shoots carrying complete flowers (Fig 1C and 1D; Table 2).

**Table 1:** *obe3-2* enhances the meristem defects of weak and intermediate *wus* alleles.

			% of phenotype (of germinated seeds)								
			seedling (10 DAG)				shoot (51DAG)				
genotype of mother plant	n	ng	wt-like	*wus-7* like	*wus-1* like	retard^1^	wt-like	*wus-6* like	*wus-1* like	disorganized leaves, no stem	arrest^2^
L*er*	244	42	99.5	0.0	0.0	0.5	99.0	0.0	0.0	0.0	1.0
*obe3-2*	51	2	98.0	0.0	0.0	2.0	98.0	0.0	0.0	0.0	2.0
*wus-1/+*	202	3	69.9	0.0	28.1	2.0	68.8	1.5	24.6	4.0	1.1
*obe3-2 wus-1/+*	205	2	75.9	0.0	20.7	3.4	69.5	3.9	1.5	19.7	5.4
*wus-6/+*	245	0	73.5	0.0	25.3	1.2	73.1	25.7	0.0	0.0	1.2
*obe3-2 wus-6/+*	212	1	74.4	0.0	24.2	1.4	74.4	0.0	0.0	23.7	1.9
*wus-7/+*	237	2	94.0	5.6	0.0	0.4	79.6	19.5	0.0	0.0	0.9
*obe3-2 wus-7/+*	228	4	93.8	4.9	0.4	0.9	75.4	19,2	0.0	4.5	0.9

Seedling phenotype classes: wt-like, shoot meristem forming a rosette of leaves; *wus-7*-like; shoot meristem termination after true leaves have been formed, *wus-1*-like: shoot meristem termination without any leaves.

Shoot phenotype classes: wt-like, rosette, indeterminate inflorescence; *wus-6*-like, disorganized leaves, indeterminate inflorescence; *wus-1*-like, disorganized leaves, stop-and-go inflorescence rarely forming flowers.

DAG, days after germination; n, number of plants analyzed; ng, not germinated;

^1^, retard: small whitish seedlings with retarded growth and cotyledons only.

^2^, arrest: plants stopped development at seedling stage.

Chi-square test results for the seedling phenotype difference between:

*wus-1*/+ vs *obe3-2 wus-1*/+, p>0.05, not significant

*wus-6*/+ vs *obe3-2 wus-6*/+, p>0.05, not significant

*wus-7*/+ vs *obe3-2 wus-7*/+, p>0.05, not significant

Chi-square test results for the shoot phenotype difference between:

*wus-1*/+ vs *obe3-2 wus-1*/+, p<0.0001, highly significant

*wus-6*/+ vs *obe3-2 wus-6*/+, p<0.0001, highly significant

*wus-7*/+ vs *obe3-2 wus-7*/+, 0.01<p<0.05, significant

**Table 2:** Flower phenotypes of *obe3-2 wus-1*, *obe3-2 wus-7* and *obe3-2 wus-6*.

Genotype	n flowers	stamens	carpels
wild type (Ler)	20	6.0±0.0	2.0±0.0
*obe3-2*	20	6.0±0.0	1.8±0.6
*wus-1*	8	1.0±0.0	0.0±0.0
*obe3-2 wus-1*	3	1.0±0.0	0–0±0.0
*wus-6*	20	1.0±0.0	0.0±0.0
*obe3-2 wus-6*	3	1.0±0.0	0.0±0.0
*wus-7*	15	5.9±0.6	1.6±0.8
*obe3-2 wus-7*	20	4.0±0.0	0.2±0.6

At 79 DAG (except the *obe3-2 wus-6* at 100DAG), opened flowers were taken from the genotyped plants and the organ numbers were counted. Organ numbers in first and second whorls were 4 sepals and 4 petals, respectively, for all genotypes.

In order to identify factors that cooperate with *WUS* in stem cell maintenance, we searched for EMS mutants that modify the stem cell defects of the intermediate allele *wus-6*. One of the isolated enhancers, *wus enhancer 9* (*wen9*), was mapped to a 97 kb region between position 5001124 and 5098789 on chromosome 1, and a nonsense mutation was identified in the predicted first exon of locus AT1G14740. The encoded protein OBERON3 (OBE3) [26], also named TITANIA1 (TTA1) [27], is 733 amino acids in size and contains a potential PHD (plant homeo domain) DNA binding domain (Fig 1A). The *wen9* mutation introduces a stop codon after the PHD domain at amino acid position 518. *OBE3* transcript levels are reduced in *wen9* to about half of the wild-type level (S1 Fig). Transformation with a 4.6 kb genomic *OBE3* fragment (Fig 1A) suppressed the enhanced phenotype of *wen9 wus-6* plants (S1 Table; S4 Fig) and two independent T-DNA insertion mutants in the *OBE3* locus enhance *wus-6* similar to *wen9*, albeit to a weaker extent (S2 Table; S5 Fig). We thus conclude that the mutation in *OBE3* caused the enhanced phenotype and assigned *wen9* as *obe3-2*.

**Fig 2:**
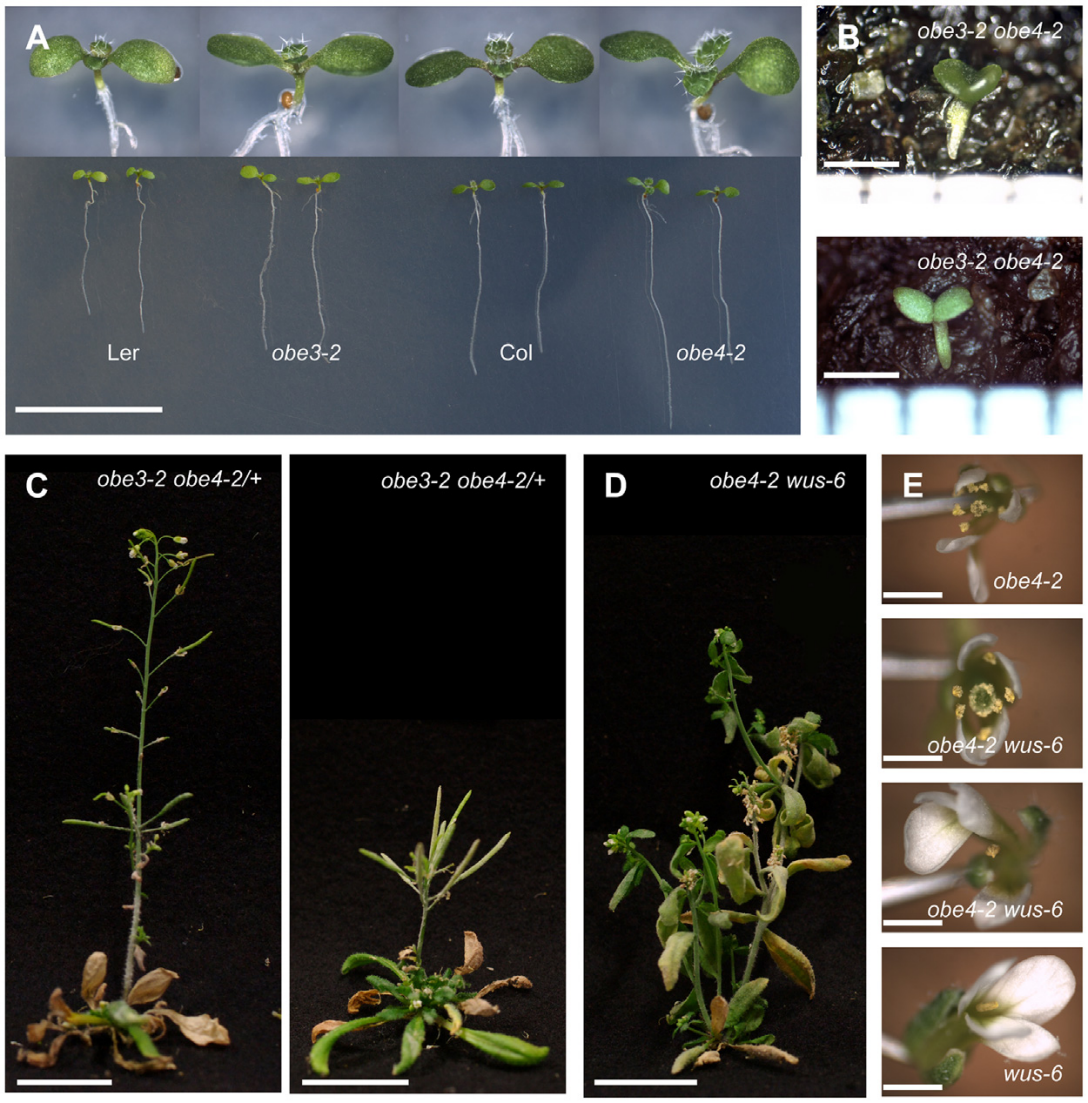
Genetic combinations of *obe3-2* and *obe4-2*. (A) Plants of *obe3-2* and *obe4-2* single mutants are indistinguishable from wild type. (B) *obe3-2 obe4-2* seedlings that did not develop further. (C) *obe3-2 obe4-2/+* plants displayed two phenotypic classes at 60 DAG. (D) *obe4-2 wus-6* plant displayed *wus-6*-like shoot formation at 60 DAG. (E) *obe4-2 wus-6* plant produced complete flowers and wus-1 like flowers. Scale bars: 2 cm (A, C and D), 1 mm (B and E).

To investigate the genetic interaction between *WUS* and *OBE3*, we analyzed double mutants between different *wus* alleles and the *obe3-2* mutant. Development of the homozygous *obe3-2* single mutant is indistinguishable from wild type (Figs 1B–1D and 2A). The *obe3-2* mutation does not affect the seedling phenotypes of *wus-1*, *-6*, or *-7* (Fig 1B; Table 1). However, it strongly reduces the postembryonic formation of inflorescence stems in all combinations (Fig 1C; Table 1) and causes the formation of leaves in a disorganized (= *wuschel*-like) pattern. In cases where flowers are made, *obe3-2* does not further enhance the already early termination of *wus-1* and *wus-6* floral meristems, but causes premature termination of *wus-7* floral meristems (Fig 1D; Table 2).

In summary, residual *WUS* activity in hypomorphic *wus* alleles requires *OBE3* for maintenance of inflorescence meristems (*wus-6* and *wus-7*) and floral meristems *(wus-7*).

### *OBE3* functions redundantly with *OBE4*

Because the *obe3-2* single mutant does not display any developmental defect, we asked whether related genes might mask its function. To this end, we isolated an insertion mutant in the closest *OBE3* homolog *OBE4*, also named *TITANIA2 (TTA2)* [27]. The *obe4-2 (SAIL_827_F11*) mutation disrupts exon1, suggesting that it is a severe loss of function allele (S2 Fig). *OBE4* transcript levels are reduced in *obe4-2* to about half of the wild-type level (S1 Fig). *obe4-2* single mutants are indistinguishable from the Col wild type (Fig 2A).

In the segregating progeny of an *obe3-2*/+ *obe4-2*/+ mother plant, we found 2.8% (n = 143) very small seedlings with partially fused cotyledons and without any true leaves, did not develop further, and were genotyped as homozygous double mutants by PCR (Fig 2B). By contrast, all *obe3-2 obe4-2/*+ plants identified by PCR (9.8%, n = 143) formed true leaves, but display severe growth retardation (Fig 2C), whereas the remaining segregating sibling plants look like wild type. We independently confirmed these results with the *obe3-1 obe4-1* combination (S3 Fig). Thus, *OBE3* and *OBE4* are redundantly required for plant growth.

*obe4-2 wus-6* double mutants are indistinguishable from *wus-6*, with the exceptions that *obe4-2 wus-6* inflorescences produced less than 10 siliques (data not shown) and that all double mutant plants (n = 6) were mosaics carrying both, wild-type-like complete flowers (40/170) and *wus-1*-like incomplete flowers (130/170; Fig 2D and 2E). Thus, in contrast to *obe3-2*, the *obe4-2* mutation restores carpel and seed development in *wus-6*, suggesting that in floral meristems, *WUS* and *OBE4* act oppositely.

### Mutual expression regulation between *WUS* and *OBE3*

**Fig 3:**
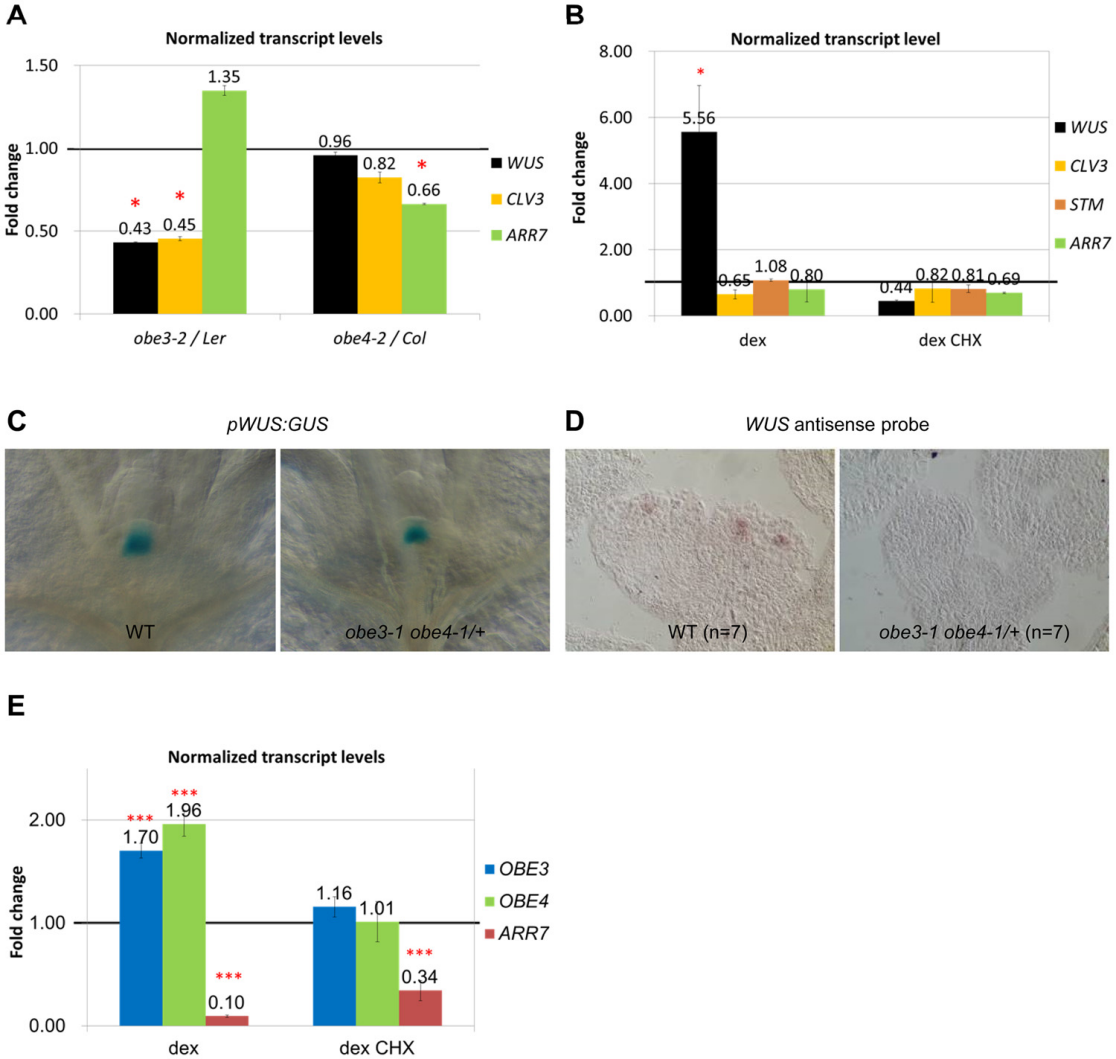
Changes of transcripts in *obe3-2* and *obe4-2*. (A) Transcript levels of 7-day-old seedlings as indicated. Error bars represent SE. (B) After induction of *OBE3* overexpression, mRNA levels of *WUS* are increased, whereas mRNA levels of *CLV3*, *STM*, and *ARR7* are unchanged in 7-day-old seedling. Error bars represent SD. (C) *pWUS*:*GUS* expression in 6-day-old *obe3-1 obe4-1/+* seedlings is confined to the OC as in the wild type. (D) *WUS* mRNA is undetectable by *in situ* hybridization in *obe3-1 obe4-1/+* floral meristems of 30-day-old plants. (E) *WUS* overexpression upregulates *OBE3* and *OBE4* mRNA levels in 7-day-old seedlings. *ARR7* expression is used as a control. Error bars represent SD. Relative mRNA levels compared to the mock control are shown.*,p<0.05, calculated from Cp’ values; ***, p<0.001, calculated from Cp’ values.

In order to investigate whether the expression levels of *WUS* and *CLV3* genes are altered, we performed qRT-PCR with the 7 day-old *obe3-2* and *obe4-2* seedlings. *WUS* and *CLV3* mRNA levels are significantly reduced in *obe3-2* (0.43 and 0.45 fold, respectively) compared to the L*er* wild type, whereas *ARR7* mRNA levels appear increased (Fig 3A). In a converse experiment, *WUS* mRNA level is increased after induction of *p35S*:*cOBE3-GR* expression and this effect is suppressed in the presence of cycloheximide, whereas mRNA levels of *CLV3*, *STM*, and *ARR7* are not significantly changed (Fig 3B). In contrast to *obe3-2*, expression levels of *WUS* or *CLV3* genes are not significantly changed in *obe4-2* (Fig 3A), but the *ARR7* mRNA level is reduced (Fig 3A). In summary, *OBE3* is required for normal *WUS* and *CLV3* expression.

Because double mutant plants are severely retarded, we analyzed the *WUS* expression pattern in *obe3-1 obe4-1/+* plants. In 6-day-old seedlings, expression of the *pWUS*:*GUS* (Fig 3C) reporter is confined to the OC of *obe3-1 obe4-1/+* plants as in the wild type. However, in 20-day-old inflorescences, *WUS* expression is not detectable in *obe3-1 obe4-1/+* (genotyped by PCR) inflorescence and floral meristems unlike in the wild type (Fig 3D).

In order to address whether *WUS* affects *OBE3/OBE4* transcript levels, we analyzed the effects of inducible WUS activity. After induction of *p35S*:*WUS-GR* plants with dexamethasone, mRNA levels of *OBE3* and *OBE4* are upregulated, and this effect is suppressed in the presence of cycloheximide (Fig 3E). The direct *WUS* target in the shoot meristem, *ARR7* [2], is used as a control for *WUS-GR* induction. Upregulation of *OBE3* and *OBE4* expression by *WUS* is also suggested by published microarray data (S3 Table). Thus, *WUS* activity is sufficient to induce *OBE3/OBE4* expression by an indirect mechanism. However, we did not detect any abnormal phenotype in *p35S*:*cOBE3* or *p35S*:*cOBE3-GR* plants.

### Overexpression of *OBE3* alleviates shoot meristem but not floral meristem defects of *wus-1*

**Fig 4:**
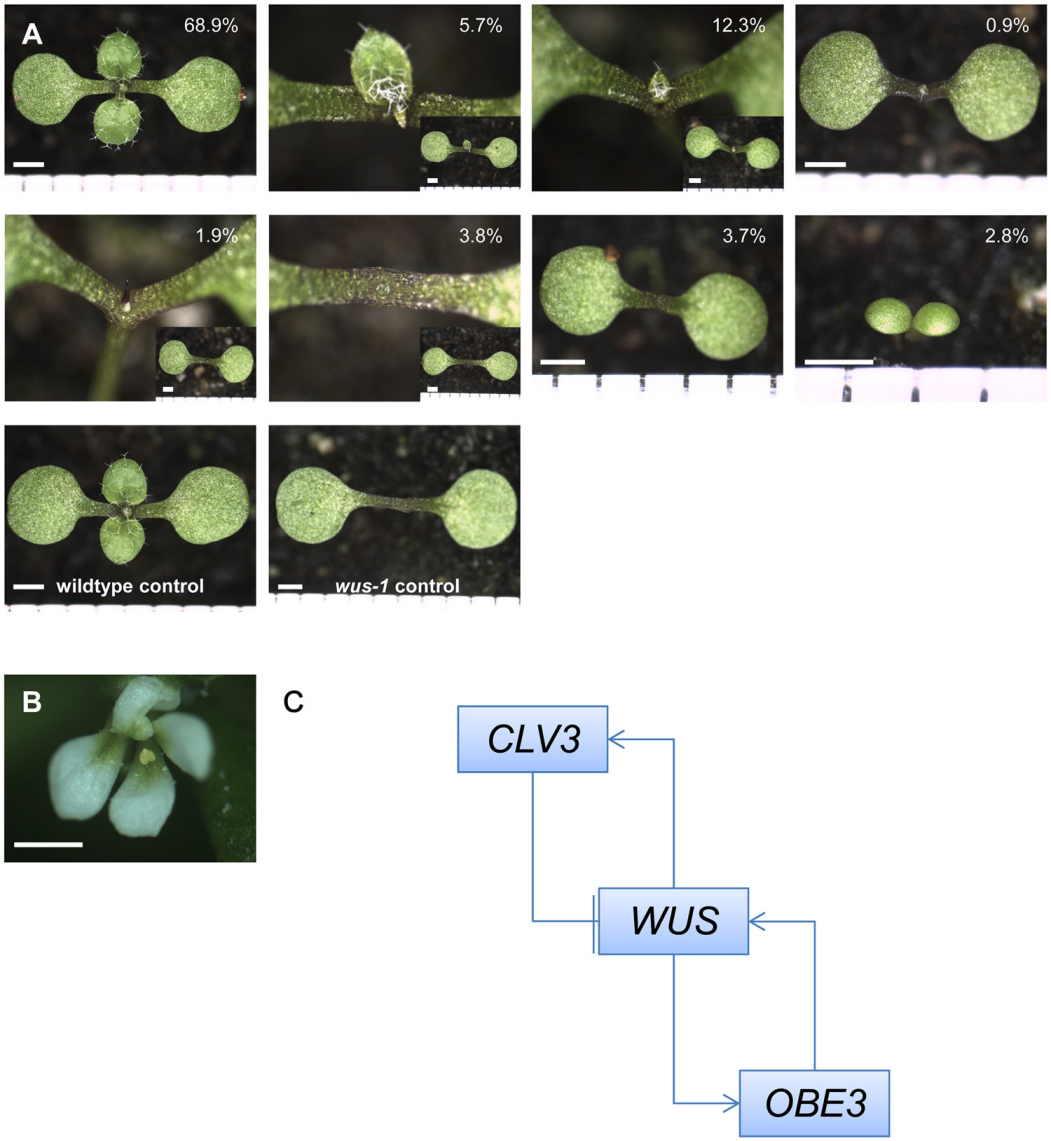
*p35S*:*cOBE3* expression partially suppresses *wus-1* defects. (A) Phenotypes of segregating seedlings in the progeny of a *p35S*:*cOBE3 wus-1/+* mother plant. (B) *p35s*:*cOBE3 wus-1* plants produce *wus-1*-like flowers. (C) Model for *WUS-OBE3* interaction. Scale bars: 1 mm.

Because *WUS* overexpression can upregulate *OBE3*, we asked whether forced expression of *OBE3* can overcome the absence of *WUS* activity and expressed *p35S*:*cOBE3* in *wus-1*. When comparing segregating *p35S*:*cOBE3 wus-1/+* plants with *wus-1* empty vector controls, we find that the number of seedlings lacking the shoot meristem are reduced (10.3% vs. 28.5%) whereas seedlings with weak shoot meristem defects are increased (20.8% vs. 6.0%; Fig 4A, S4 Table). Furthermore, postembryonic shoot formation is increased (29.2% vs. 14.7%; S4 Table). The difference between *p35S*:*cOBE3 wus-1/+* and empty vector *wus-1/+* control is highly significant in both seedling and shoot stages (both Chi square, p<0.0001). By contrast, defective *wus-1* flower development is not altered by *p35S*:*cOBE3* (Fig 4B). Thus, *OBE3* activity can partially replace *WUS* activity in seedling and inflorescence shoot meristems, but not in the floral meristem.

## Discussion

Stem cell homeostasis requires the balanced activities of a complex network of regulatory factors. Despite strong advances, our knowledge of regulatory pathways is still fragmentary with many components unknown. This is due in part to the fact that a limited number of mutants display informative stem cell phenotypes. Furthermore, many other essential stem cell factors may remain undiscovered due to genetic redundancy or pleiotropic mutant phenotypes. Here we used a modifier screen to overcome this problem and discovered the *obe3-2* mutant as an enhancer of the hypomorphic *wus-6* allele.

### What is the developmental nature of the *WUS-OB3* interaction?

The role of *WUS* in stem cell maintenance can be observed at several developmental stages. Mature *wus-1* embryos and seedlings lack shoot meristem stem cells and display differentiated cells instead. Postembryonically formed adventitious meristems terminate prematurely after forming a few leaves. Only occasionally an inflorescence is formed, but it terminates prematurely after formation of 1–3 flowers, which in turn terminate prematurely after a single first anther. Whereas seedling and floral meristems appear to absolutely require *WUS* activity, the occasional formation of inflorescences suggests that at this stage other factors can sustain stem cells for some time [21]. Although *OBE3* is ubiquitously expressed [27], the *obe3-2* mutation enhances only the premature vegetative shoot meristem termination in *wus-1* and thus represents one of these additional factors. In the hypomorphic allele *wus-6*, *obe3-2* causes premature termination of the inflorescence meristem indistinguishable to *wus-1*. Finally, in combination with *wus-7*, which as a single mutant displays higher floral meristem activity compared to *wus-1* and *wus-6*, *obe3-2* enhances premature termination of the floral meristem. These results indicate that in addition to the seedling phase, *OBE3* is required for residual WUS activity of hypomorphic *wus* alleles in inflorescence and floral meristems. By contrast, *obe3-2* does not enhance *wus* defects in embryonic shoot meristem formation.

Curiously, despite their redundancy in the shoot meristem, *obe3* enhances *wus* flower termination whereas *obe4* mutant suppresses it. One possible reason for this particular behavior might be that in *obe3-1* and *obe3-2* mutants, the C-terminal region of *OBE3* is disrupted and the PHD domain is still intact, whereas in *obe4-1* and *obe4-2* mutants the PHD domain is disrupted. Alternatively, both wild-type proteins might have divergent functions specifically in floral meristems.

Considering the ubiquitous expression of *OBE3*, it is noteworthy that the *obe3-2* mutation reduces the organ number only of the two inner whorls of *wus-7* but does not affect the perianth. A plausible explanation is that *WUS*-mediated stem cell maintenance is only required to provide the cells for the inner two whorls, whereas perianth organs appear to consume the cells present in the initial floral meristem formed independently of *WUS*, as described previously [6].

### What is the genetic nature of the *WUS-OB3* interaction?

Based on our mutant analysis and expression studies, *OBE3* appears to act downstream of *WUS*. On the other hand, *WUS* expression levels are reduced in *obe3-2* mutants and increased by *OBE3* overexpression from the ubiquitous 35S promoter. The reduction of *pWUS*:*GUS* expression in the shoot meristem of *obe3-1 obe4-1/+* mutants and the requirement of *OBE3* in *wus* hypomorphs suggest that this is also the case in shoot meristem regulation. One plausible interpretation of this data is that *WUS* and *OBE3* reinforce each other's expression in a positive feedback loop (Fig 4C), albeit this effect seems moderate.

OBE3 is a member of a small group of related proteins and, together with its closest homolog OBE4, is redundantly required for plant growth, consistent with previous observations [27]. The seedling lethality of *obe3-2 obe4-2* double mutants suggests that both genes are involved in several processes other than shoot meristem regulation. Likewise, *obe1 obe2* [28] displays seedling lethality, but not any other *obe* double mutant combinations, indicating two pairs of redundant functions in this group, OBE1,2 and OBE3,4. In contrast to *obe3-2 wus-6* plants, *obe4-2 wus-6* double mutants have *wus-6*-like inflorescences and partially restored floral organs and seed production. This indicates that *OBE3* and *OBE4*, in addition to their redundant functions in general growth control, have opposite roles at least in floral meristems.

### What is a possible molecular basis of interaction?

The upregulation of *OBE3* mRNA levels by *WUS* and vice versa is abolished by the presence of the protein inhibitor cycloheximide, suggesting that intermediate components are necessary. OBEs are PHD domain containing proteins, which originally were found by their homology to the Potyvirus VPg-interacting protein [29]. The PHD domain is reported to bind to potentially activating H3K4me2/3 modifications [30–34]. Further experiments are necessary to address how OBE affects *WUS* expression and vice versa and whether the enhancement of hypomorphic *wus* mutant defects by *obe3-2* can be attributed to the reduction of *WUS* expression levels.

## Material and Methods

### Plant materials and growth conditions

The *obe3-2* mutant was isolated from EMS-mutagenized populations in a *wus-6* background in L*er* [25]. The insertion alleles *obe3-3*^*SALK_078036*^, *obe3-4*^*SALK_042597c*^ and *obe4-2*^*SAIL_827_F11*^ in the Col background were identified from the SALK collection [35] of T-DNA tagged lines and the SAIL collection [36], respectively. All other mutant alleles and transgenic lines used in this study are listed in S5 Table. For segregation analysis, entire siliques from genotyped mother plants were harvested and the seeds were sown in randomized schemes [37]. Plant growth conditions were as previously described [6]

### Mapping, genetic analysis and PCR genotyping

The *wen9 wus-6/+* x Col cross was performed for map-based cloning. Among 12113 F2 plants from F1 parents genotyped as *wus-6*/+, we identified 446 plants (3.7%) with an enhanced phenotype. The *wen9* mutation was mapped with SSLP and dCAPS markers to a 97 kb region in chromosome 1 (between SNP CER465614 and CER424346). Sequencing of all 23 candidate loci in this region detected a mutation only in the *OBE3* gene. The identified G1554>A “stop” mutation is in exon1 of the predicted reading frame of locus AT1G14740. The primers wen9-F (5’- CAGAGATGTTTGGATTCGTTAAGGATGTTTTTGTGTGTTGCGCTAAGAATCG-3’) and wen9-R (5’-GAAATTGTGATAAGAGAAGG-3’) were used for genotyping PCRs (Ta 55°C). After TaqI restriction cleavage, the wild type displays a 300bp band, and *wen9* displays a 250bp and a 50bp band. Genotyping primers of other mutants including T-DNA lines used in this study are listed in S6 Table.

### Preparation of constructs and selection of transformants

The genomic fragment including the intergenic region of *OBE3* was amplified by PCR from Ler and cloned as *pOBE3*:*gOBE3* in a *pGPTV-HPT*-based vector. The RALF11-03K20 cDNA clone from RIKEN BRC was used to amplify the full length cDNA by PCR for construct preparation. The cDNA fragments starting from ATG to the end of gene, with or without the stop codon, were cloned as: *p35S*:*cOBE3*, and *p35S*:*cOBE3-GR* respectively, in a *pGreenII*-based vector. *Arabidopsis* plants were transformed by floral dip, and T1 seeds were selected on plates with the respective antibiotics.

### Quantitative RT-PCR analysis

Arabidopsis seeds were surface-sterilized with 1% hypochlorite for 10 minutes and washed two times with 70% ethanol. Sterilized seeds were sown on 1/2 MS plates, stratified for 3 days in the dark at 4°C and then grown in a Percival growth cabinet with constant illumination for 7 or 10 days. For all qRT-PCR experiments, 3 biological replicates with two technical replicates each were done.

For experiments without further treatment, seedlings were collected from the plates and frozen in liquid nitrogen immediately. For the induction experiments, dexamethasone (5 μM), with or without cycloheximide (50 μM) were applied by spraying the plates, and flooding the seedlings for 15 minutes. After removal of the liquid, seedlings were returned to the Percival growth cabinet for 4 hours before sample collection.

Total RNA was extracted from whole seedlings using the RNeasy^®^ Mini kit (QIAGEN), followed by RQ1 RNase-Free DNase (Promega) treatment and reverse-transcribed with SuperScript III First-Strand Synthesis SuperMix for qRT-PCR (Invitrogen). Quantitative PCR was performed with the LightCycler^®^ 480 system (Roche) coupled with SYBR Green I Master (Roche). For each qPCR reaction, 25 ng of cDNA was used. Transcript level analysis was carried out according to a published protocol [38]. For statistical analysis, ANOVA or *t*-tests were performed on Cp’ values. The Cp’ values were calculated after normalizing the Cp values with three independent reference genes, which passed the geNorm v3.5 [39] test. For graphic presentation, Normalized Relative Quantity (NRQ) was first rescaled by setting NRQ of mock treated wild-type samples as 1, then adjusted for the unspecific DEX effect and the transgene effect sequentially in order to calculate the transcript fold change. Fold changes were plotted as bar graphs. Primers used for quantitative PCR are listed in S7 Table.

## Supporting Information

S1 Fig Transcript level of *OBE3* and *OBE4* is reduced in the corresponding mutants. (TIF)

S2 Fig Structure of *OBE4*. (TIF)

S3 Fig Genetic combinations of *obe3-1* and *obe4-1*. (TIF)

S4 Fig A genomic *OBE3* fragment suppresses the enhanced phenotype of *obe3-2 wus-6*. (TIF)

S5 Fig *OBE3* T-DNA insertion mutants enhance *wus-6* defects. (TIF)

S1 Table A genomic *gOBE3* fragment suppresses the effects of the *wen9* mutation. (PDF)

S2 Table *OBE3* T-DNA insertion lines enhance *wus-6*. (PDF)

S3 Table Microarray expression levels of *OBE* genes. (PDF)

S4 Table Phenotypes of segregating *p35S*:*cOBE3 wus-1/+* plants. (PDF)

S5 Table Mutants and Transgenic lines used in this study. (PDF)

S6 Table Primers used for genotyping mutant alleles. (PDF)

S7 Table Primers used for qPCR. (PDF)
