# Supplementary figures and images for: OBE3 and WUS Interaction in Shoot Meristem Stem Cell Regulation
Source: PLoS One. 2016 May 19;11(5):e0155657. doi: 10.1371/journal.pone.0155657 (PMC4873020; doi:10.1371/journal.pone.0155657)

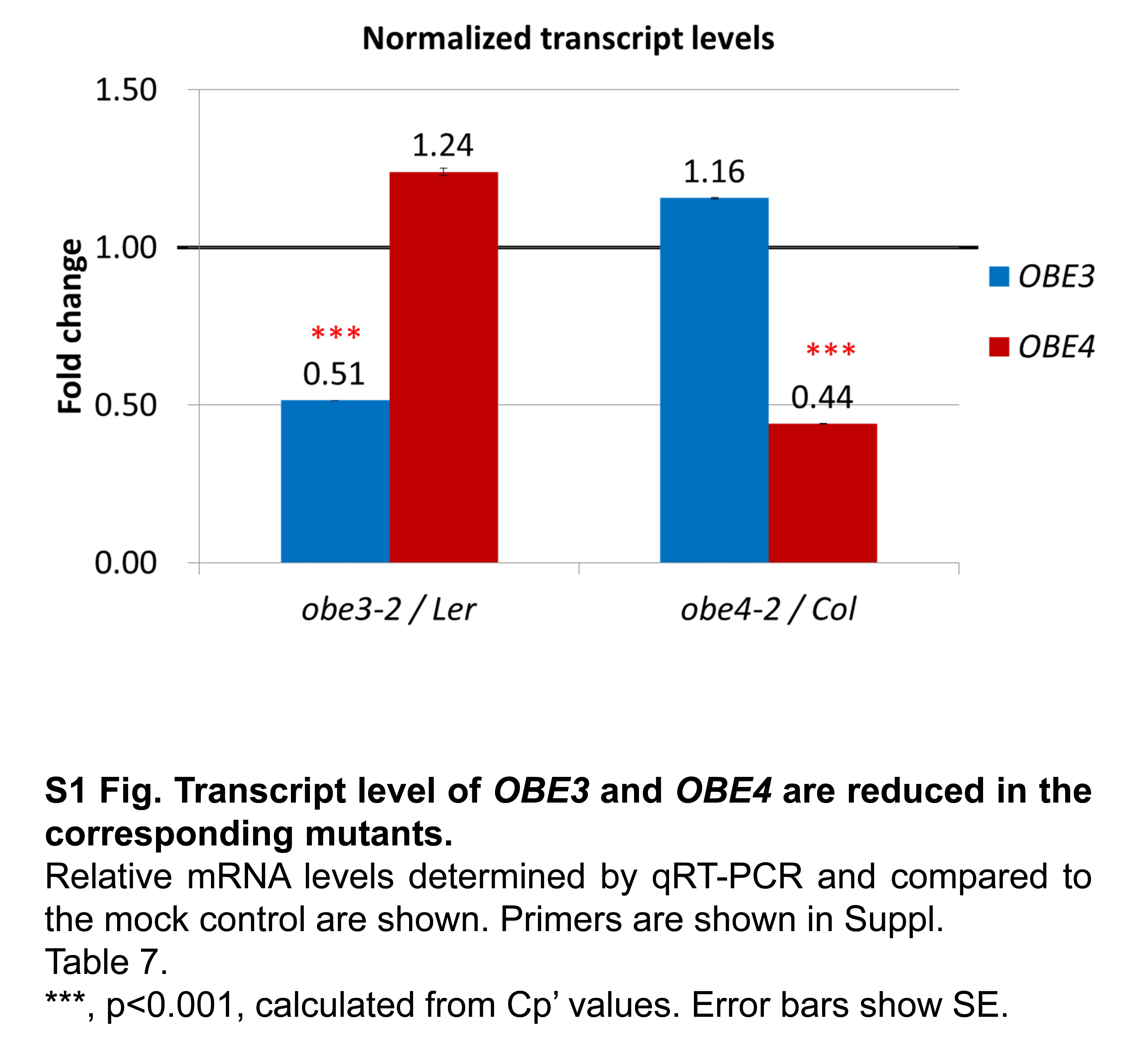

Supplement: S1 Fig — (TIF) [file pone.0155657.s001.tif]

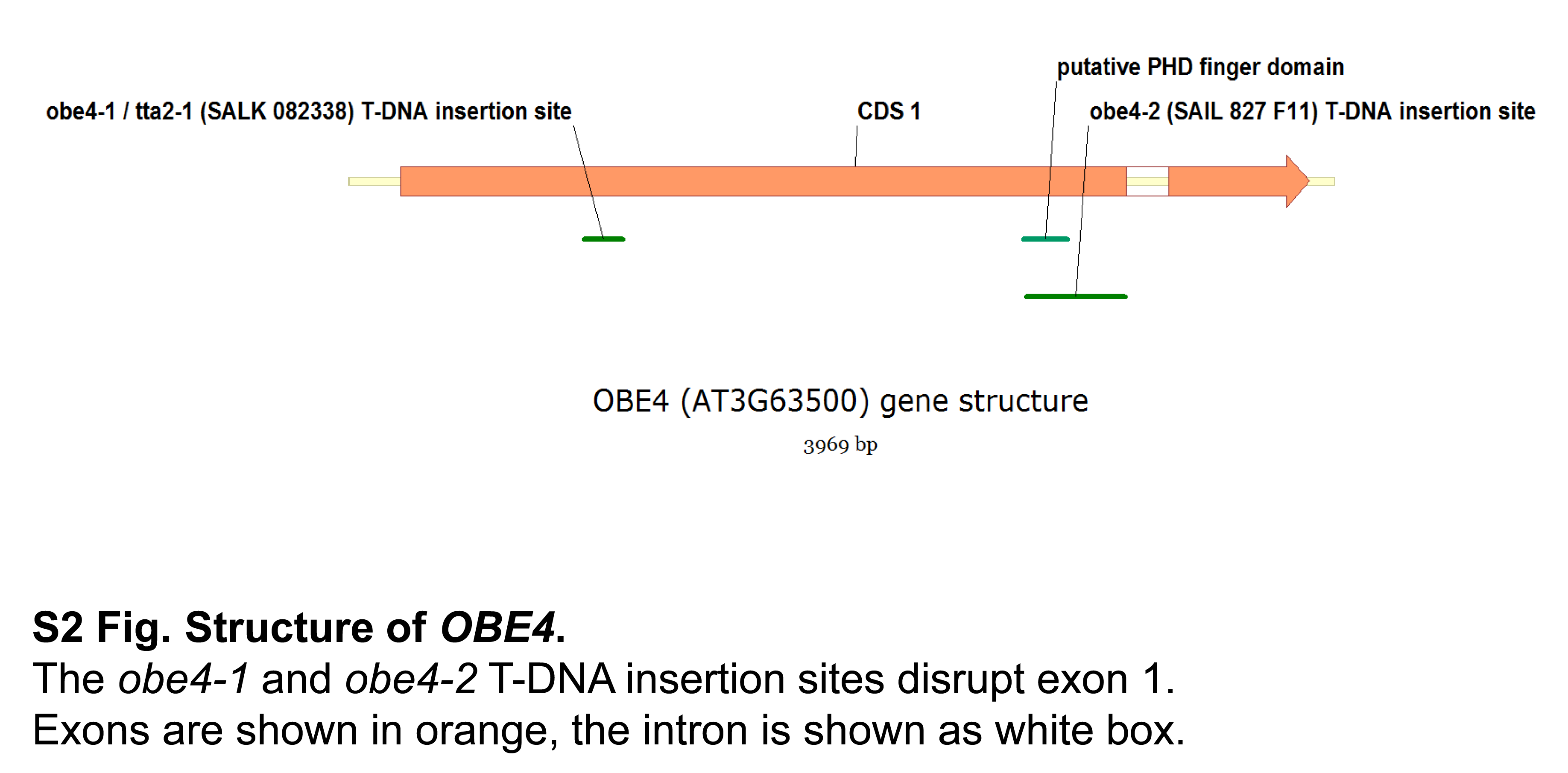

Supplement: S2 Fig — (TIF) [file pone.0155657.s002.tif]

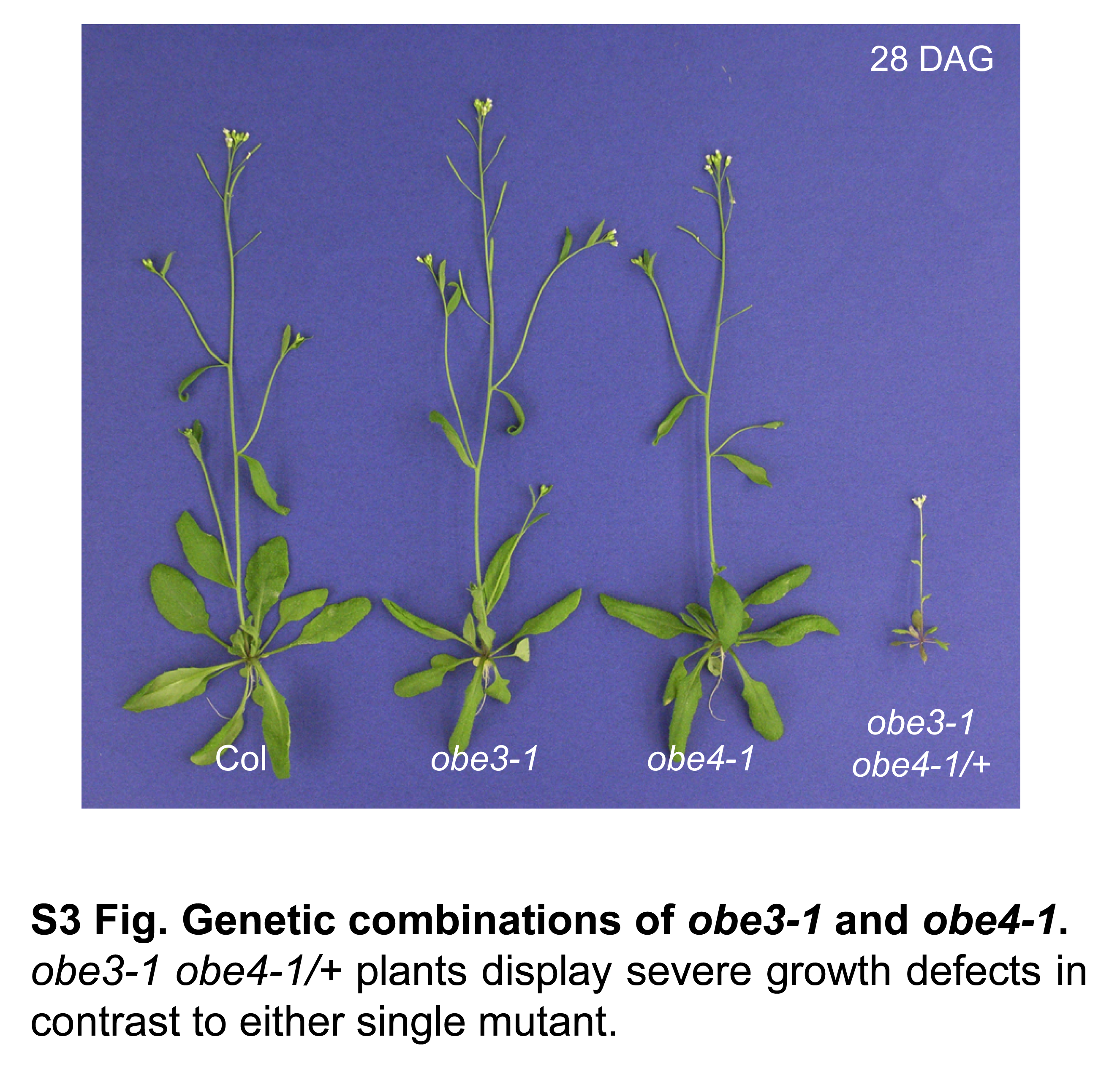

Supplement: S3 Fig — (TIF) [file pone.0155657.s003.tif]

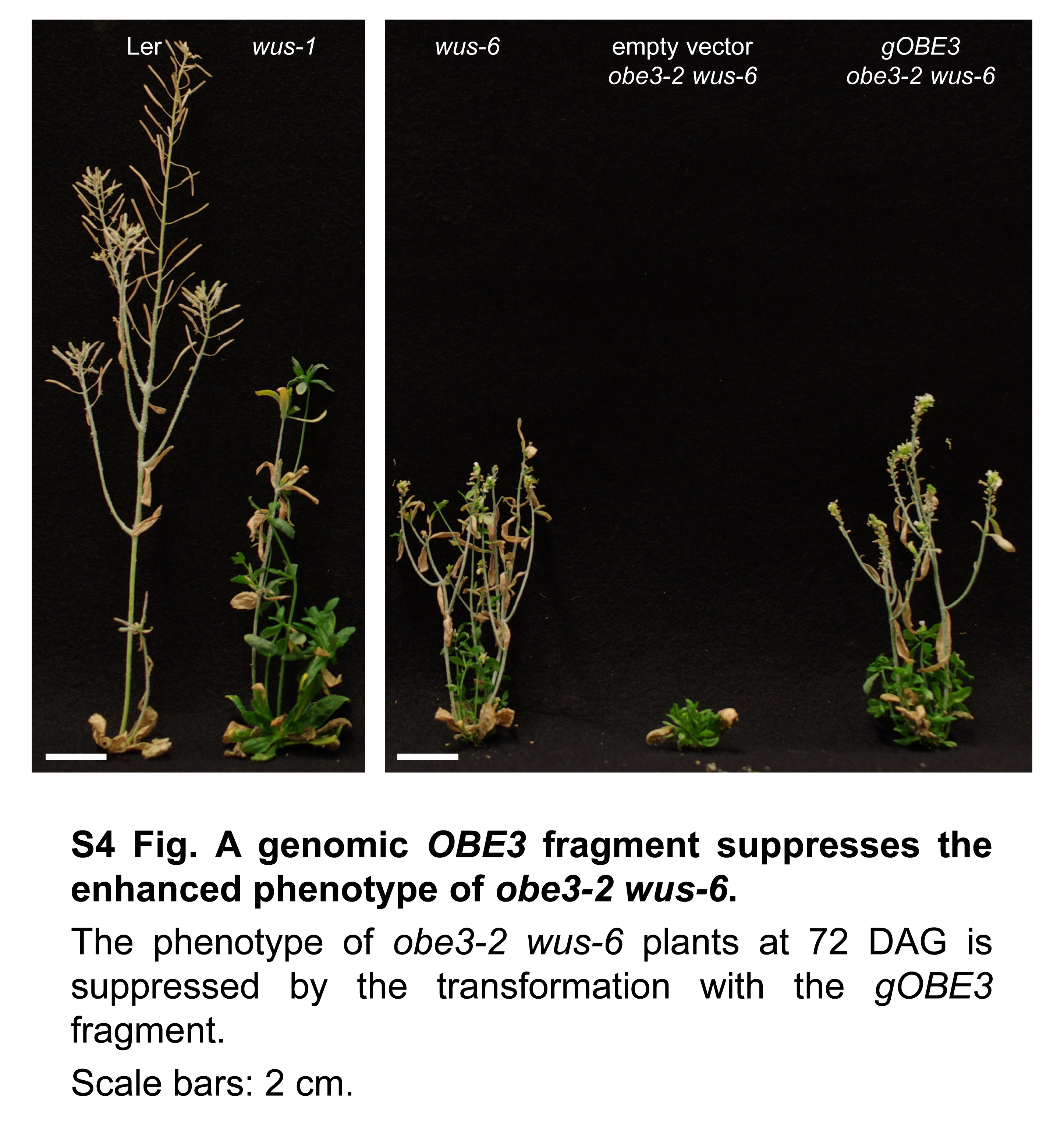

Supplement: S4 Fig — (TIF) [file pone.0155657.s004.tif]

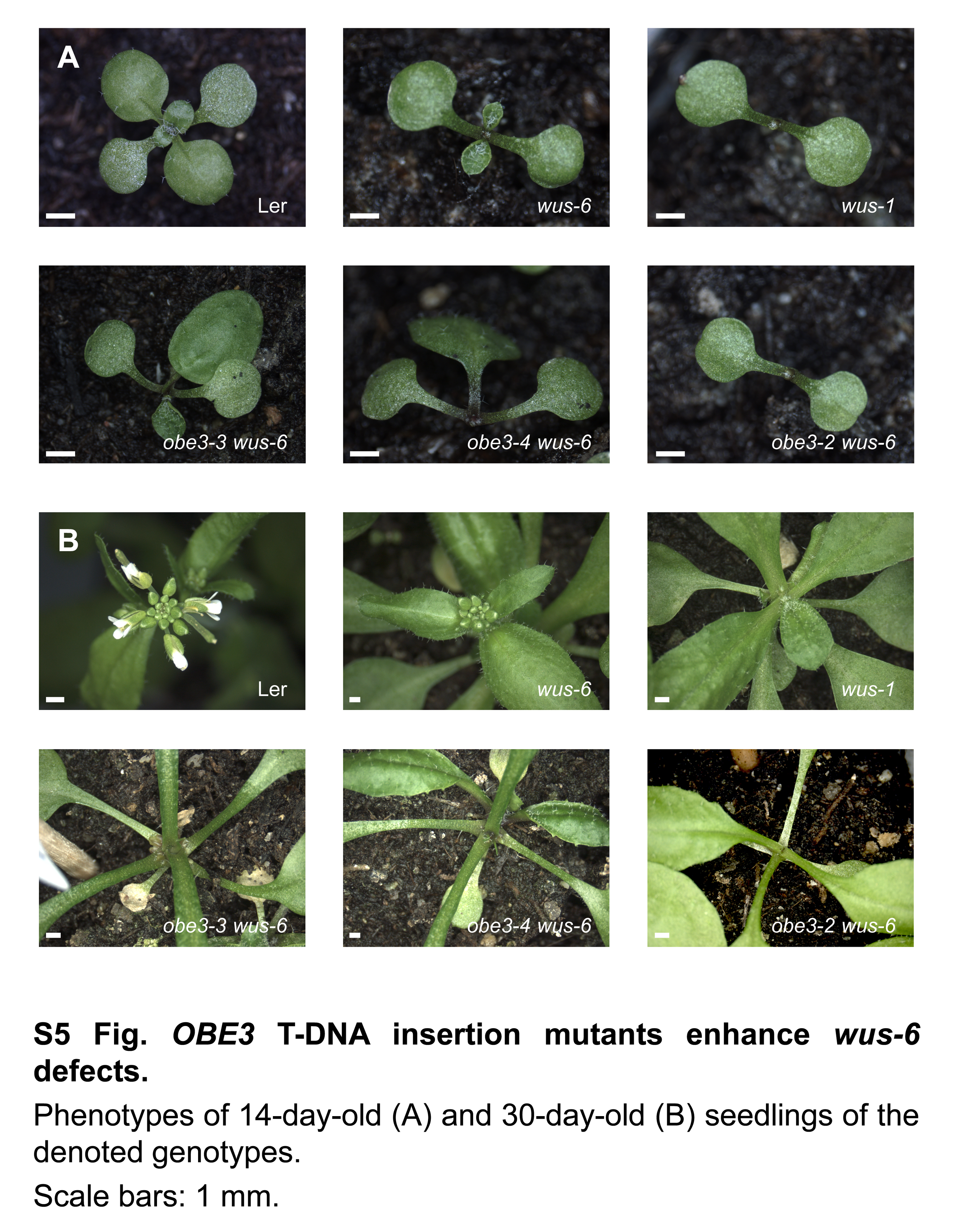

Supplement: S5 Fig — (TIF) [file pone.0155657.s005.tif]
