# Supplementary material for: OBE3 and WUS Interaction in Shoot Meristem Stem Cell Regulation
Source: PLoS One. 2016 May 19;11(5):e0155657. doi: 10.1371/journal.pone.0155657 (PMC4873020; doi:10.1371/journal.pone.0155657)
Supplement: S1 Table — (PDF) [file pone.0155657.s006.pdf]

S1 Table. A genomic *gOBE3* fragment suppresses the effects of the *wen9* mutation.

Construct	n	% of the transformants		
		wt-like	no shoot	*wus-6*-like
gOBE3	56	66.1	0.0	33.9
Control, empty vector	22	77.3	22.7	0.0

T1 seedlings of transformed *wus-6/+* mother plants were selected on hygromycin supplemented media for 12 days and then transferred to soil for phenotypic analysis at 30 DAG.

wus-6 like: indeterminate shoot but prematurely terminated floral meristems
