# Supplementary material for: OBE3 and WUS Interaction in Shoot Meristem Stem Cell Regulation
Source: PLoS One. 2016 May 19;11(5):e0155657. doi: 10.1371/journal.pone.0155657 (PMC4873020; doi:10.1371/journal.pone.0155657)
Supplement: S2 Table — (PDF) [file pone.0155657.s007.pdf]

S2 Table. ***OBE3*** **T-DNA insertion lines enhance** ***wus-6.***

genotype of mother	N	% of plants with disorganized leaves, no stem at 30 DAG
L*er*	20	0.0
wus-1/+	8	25.0
wus-6/+	10	0.0
obe3-2	10	0.0
obe3-2 wus-6/+	11	36.4
obe3-3	10	0.0
obe3-3/+ wus-6/+	36	11.1
obe3-4	10	0.0
obe3-4/+ wus-6/+	42	7.1

The T-DNA insertion mutants *obe3-3* and *obe3-4* were crossed to *wus-6/+*, and the phenotypes of segregating F2 seedlings were analyzed together with the single mutants at 30 days after germination.
