# Supplementary material for: OBE3 and WUS Interaction in Shoot Meristem Stem Cell Regulation
Source: PLoS One. 2016 May 19;11(5):e0155657. doi: 10.1371/journal.pone.0155657 (PMC4873020; doi:10.1371/journal.pone.0155657)
Supplement: S3 Table — (PDF) [file pone.0155657.s008.pdf]

S3 Table. Microarray expression levels of *OBE* genes.

	Average expression levels of overexpression lines[1]				Average expression level in different domains[2]			Putative WUS binding motif[3]	WRS list[3]
gene	*WUS*	*STM*	*LFY*	*GUS*	*CLV3*	*WUS*	*FIL*		
OBE1	1478	1425	1181	1750	1150	1047	762	yes	no
OBE2	n/a	n/a	n/a	n/a	n/a	n/a	n/a	yes	no
OBE3	1747	1665	1491	1378	410	645	306	yes	no
OBE4	4424	3837	5130	3622	1237	1450	1256	no	no
WUS	28339	194	215	189	1754	1095	29		induced
CLV3	428	392	370	395	968	67	5		repressed

Summarized from: [1],[1]; [2],[2]; [3],[3]
OBE2 is not present on Affymetrix ATH1 microarrays.

References:

1. Leibfried A, To JP, Busch W, Stehling S, Kehle A, Demar M, et al. WUSCHEL controls meristem function by direct regulation of cytokinin-inducible response regulators. Nature. 2005;438(7071):1172-5. PubMed PMID: 16372013.
2. Yadav RK, Girke T, Pasala S, Xie M, Reddy GV. Gene expression map of the Arabidopsis shoot apical meristem stem cell niche. Proc Natl Acad Sci U S A. 2009;106(12):4941-6. PubMed PMID: 19258454.
3. Busch W, Miotk A, Ariel FD, Zhao Z, Forner J, Daum G, et al. Transcriptional Control of a Plant Stem Cell Niche. Developmental Cell. 2011;18(5):841-53.
