# Supplementary material for: OBE3 and WUS Interaction in Shoot Meristem Stem Cell Regulation
Source: PLoS One. 2016 May 19;11(5):e0155657. doi: 10.1371/journal.pone.0155657 (PMC4873020; doi:10.1371/journal.pone.0155657)
Supplement: S4 Table — (PDF) [file pone.0155657.s009.pdf]

S4 Table. Phenotypes of segregating *p35S:cOBE3 wus-1/+* plants.

genotype of mother plant	n	ng	% of phenotype (of germinated seeds)						
			seedling 10 DAG (****)			shoot 21 DAG (****)			
			wt-like	weak SAM defect	strong SAM defect	wt-like	stop and go	no stem	dead
p35S:cOBE3 wus-1/+	106	0	68.9	20.8	10.3	70.8	29.2	0.0	0.0
NK43 wus-1/+	119	3	65.5	6.0	28.5	62.9	14.7	17.2	5.2

Entire siliques from mother plants were sown on corresponding antibiotic selection plates to confirm the homozygosity of the transgene. Three entire siliques from the confirmed plants were then sown on soil and phenotypes were recorded at 10 DAG and 21 DAG.
NK43. empty vector;
Seedling phenotype classes: weak defect, seedling shoot meristems terminates with 1-2 leaves or filaments formed; strong defect, seedling shoot meristems terminates without any leaf formed;
Shoot phenotype classes: wt-like, indeterminate inflorescence and complete flowers; stop-and-go, repeatedly initiated shoot meristems terminate prematurely after a few organs have been formed, incomplete flowers;
DAG, days after germination; n, number of plants analyzed; ng, not germinated;
The difference between *p35S:cOBE3 wus-1*/+ and the control is highly significant in both seedling (****, $p<0.0001$) and shoot (****, $p<0.0001$) stages
