# Supplementary material for: OBE3 and WUS Interaction in Shoot Meristem Stem Cell Regulation
Source: PLoS One. 2016 May 19;11(5):e0155657. doi: 10.1371/journal.pone.0155657 (PMC4873020; doi:10.1371/journal.pone.0155657)
Supplement: S5 Table — (PDF) [file pone.0155657.s010.pdf]

S5 Table. Mutants and Transgenic lines used in this study.

Plant lines	Ecotype background	Reference	T-DNA backbone and selection marker
wus-1	Ler	[4]	
wus-6	Ler	[5]	
wus-7	Ler	[6]	
p35S:WUS-GR	Ler	[7]	
obe3-1 / tta1-1 (SALK_042597)	Col	[8] [9]	
obe3-3(SALK_078036)	Col	[8]	
obe3-4(SALK_042597c)	Col	[8]	
obe4-1 / tta2-1 (SALK_082338)	Col	[8] [9]	
obe4-2(SAIL_827_F11)	Col	[10]	
obe3-2(wen9)	Ler	This study	
pOBE3:gOBE3	Ler	This study	*pGPTV-HPT*, hygromycin
p35S:cOBE3	Ler	This study	*pGreenII*, norflurazon
p35S:cOBE3-GR	Ler	This study	*pGreenII*, norflurazon

References:

4. Laux T, Mayer KFX, Berger J, Jürgens G. The *WUSCHEL* gene is required for shoot and floral meristem integrity in Arabidopsis. Development. 1996;122:87-96.

5. Hamada S, Onouchi H, Tanaka H, Kudo M, Liu Y-G, Shibata D, et al. Mutations in the *WUSCHEL* gene of *Arabidopsis thaliana* result in the development of shoots without juvenile leaves. the Plant Journal. 2000;24(1):91-101.

6. Graf P, Dolzblasz A, Würschum T, Lenhard M, Pfreundt U, Laux T. *MGOUN1* Encodes an *Arabidopsis* Type IB DNA Topoisomerase Required in Stem Cell Regulation and to Maintain Developmentally Regulated Gene Silencing. The Plant Cell. 2010;22:716-28.

7. Lenhard M, Jürgens G, Laux T. The *WUSCHEL* and *SHOOTMERISTEMLESS* genes fulfil complementary roles in *Arabidopsis* shoot meristem regulation. Development. 2002;129:3195-206.

8. Alonso JM, Stepanova AN, Leisse TJ, Kim CJ, Chen H, Shinn P, et al. Genome-Wide Insertional Mutagenesis of *Arabidopsis thaliana*. Science. 2003;301(5633):653-7.

9. Saiga S, Möller B, Watanabe-Taneda A, Abe M, Weijers D, Komeda Y. Control of embryonic meristem initiation in Arabidopsis by PHD-finger protein complexes. Development. 2012;139(8):1391-8.

10. Sessions A, Burkea E, Prestinga G, Auxb G, McElverb J, Pattonb D, et al. A High-Throughput Arabidopsis Reverse Genetics System. The Plant Cell. 2002;14:2985-94.
