# Supplementary material for: OBE3 and WUS Interaction in Shoot Meristem Stem Cell Regulation
Source: PLoS One. 2016 May 19;11(5):e0155657. doi: 10.1371/journal.pone.0155657 (PMC4873020; doi:10.1371/journal.pone.0155657)
Supplement: S6 Table — (PDF) [file pone.0155657.s011.pdf]

S6 Table. Primers used for genotyping mutant alleles.

Genotyping	Type	Primers	Fragment	Ta (°C)
wus-1	dCAPS (BsaBI)	FOR: TAGTATGGTCTGGATTCTGGAATC<br>REV: TATTTGTATTAATGAATTATAGTTTGATACGTA	WT 193 bp<br>Mut 160 bp + 33 bp	55
wus-7	dCAPS (XmnI)	FOR: CCG ACC AAG AAA GCG GCA ACA<br>REV: AGACGTTCTTGCCCTGAATCTTT	WT 216 bp<br>Mut 193 bp + 23 bp	55
wus-6	PCR	FOR1 (WT): TGACAAGAGAATTTCCTTCC<br>FOR2 (Mut): AGGAGATGCACTGATTTATC<br>REV: GAGATAGTAAGGTATTGAGT	WT 258 bp<br>Mut 350 bp	55
obe3-2 (wen9)	dCAPS (TaqI)	FOR: CAGAGATGTTTGGATTCGTTAAGGATGTTT-TTGTGTGTTGCGCTAAGAATCG<br>REV: GAAATTGTGATAAGAGAAGG	WT 300 bp<br>Mut 250 bp + 50 bp	55
SALK_078036 obe3-3 T-DNA insertion line	PCR	FOR1(LBa1): TGGTTCACGTAGTGGGCCATCG<br>FOR2: GTGCAAATAAATATACGATT<br>REV: GATGAGTTTGGTTGTTTAGA	WT 870 bp<br>Mut 670 bp	55
SALK_042597c obe3-4 T-DNA insertion line	PCR	FOR1(LBb1): GCG TGG ACC GCT TGC TGC AAC T<br>FOR2: TGTTTCATTGCATTGGATGT<br>REV: AGACAATTTCCAGAGAAACA	WT 932 bp<br>Mut 600 bp	55
SAIL_827_F11 obe4-2 T-DNA insertion line	PCR	FOR1 (SAIL LB): TTCATAACCAATCTCGATACAC<br>FOR2: TGATATAACACTGGAATTGC<br>REV: TCACAGTCTGCAATCAACAG	WT 800 bp<br>Mut 650 bp	50
