# Supplementary material for: OBE3 and WUS Interaction in Shoot Meristem Stem Cell Regulation
Source: PLoS One. 2016 May 19;11(5):e0155657. doi: 10.1371/journal.pone.0155657 (PMC4873020; doi:10.1371/journal.pone.0155657)
Supplement: S7 Table — (PDF) [file pone.0155657.s012.pdf]

S7 Table. Primers used for qPCR.

Primer Name		Oligo sequence
Ref 1 At4g34270 F	oIR104	GAACTGGCTGACAATGGAGTG
Ref 1 At4g34270 R	oIR105	ATCAACTCTCAGCCAAAATCG
Ref 2 At2g28390 F	oIR106	AACTCTATGCAGCATTTGATCCACT
Ref 2 At2g28390 R	oIR107	TGATTGCATATCTTTATCGCCATC
Ref 3 At4g26410 F	oIR112	GAGCTGAAGTGGCTTCCATGAC
Ref 3 At4g26410 R	oIR113	GGTCCGACATACCCATGATCC
WUS F	oIR102	AACCAAGACCATCATCTCTATCATC
WUS R	oIR103	TCAGTACCTGAGCTTGCATGA
CLV3 F	oSB324	GTTCAAGGACTTTCCAACCGCAAGATGAT
CLV3 R	oSB325	CCTTCTCTGCTTCTCCATTTGCTCCAACC
STM F	oTF201	ACAACTGCTTGATTGGTGGAGCC
STM R	oTF202	TCTGGTCCAGCCCCGTTGAT
ARR7 F	ADp20	GCATTCAGAGAAGTACCAGTAGTG
ARR7 R	ADp21	GCTAAGGTCTTGGCCTCTATAC
WEN9/OBE3 F	oTF162	CTGGATTGTTGAAGAGAATGGA
WEN9/OBE3 R	oTF163	AGCAGAGTGCAGAGAAATGG
OBE4 F	oTF196	TGATAGGCCTTCCTGAAGC
OBE4 R	oTF197	CGTATATGAACGGTGCACTAGTT
